# Supplementary figures and images for: Human pericytes degrade diverse α-synuclein aggregates
Source: PLoS One. 2022 Nov 18;17(11):e0277658. doi: 10.1371/journal.pone.0277658 (PMC9674377; doi:10.1371/journal.pone.0277658)

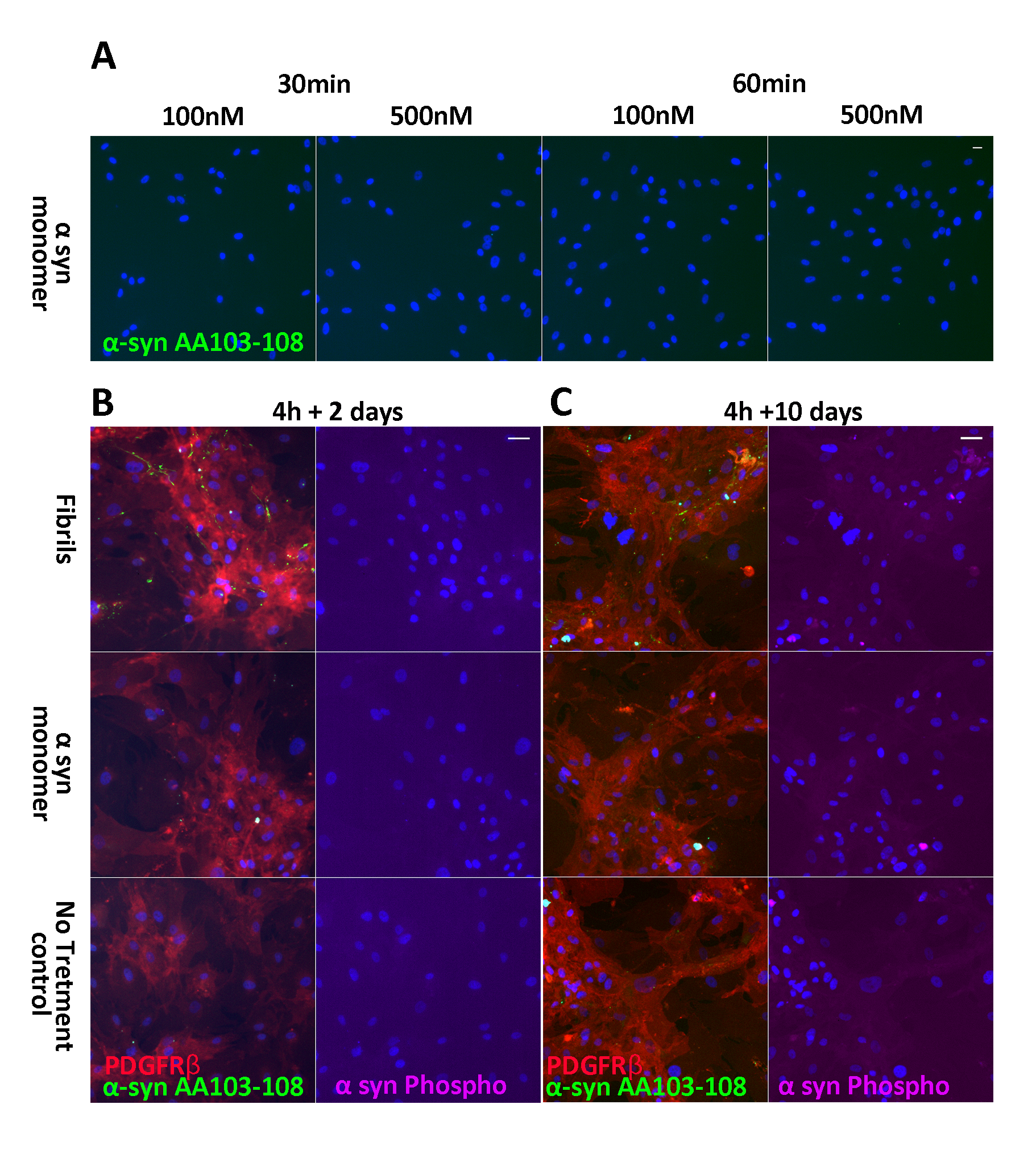

Supplement: S1 Fig — No α-syn puncta detected with α-syn specific antibodies AA103-108 (green) after treatment with monomeric α-syn (100μM and 500μM) (A). Immunofluorescent labelling of endogenous, monomeric or fibrillar α-syn treated pericytes with α-syn specific antibodies AA103-108 (green), PDGFRβ (red) and α-syn Phospho S129 (magenta) after 4 hour pre-treatment with 100nM Fibrils or monomeric α-syn (2 days, B; 10 days C). Scale bars represent 20 μm. (TIF) [file pone.0277658.s001.tif]

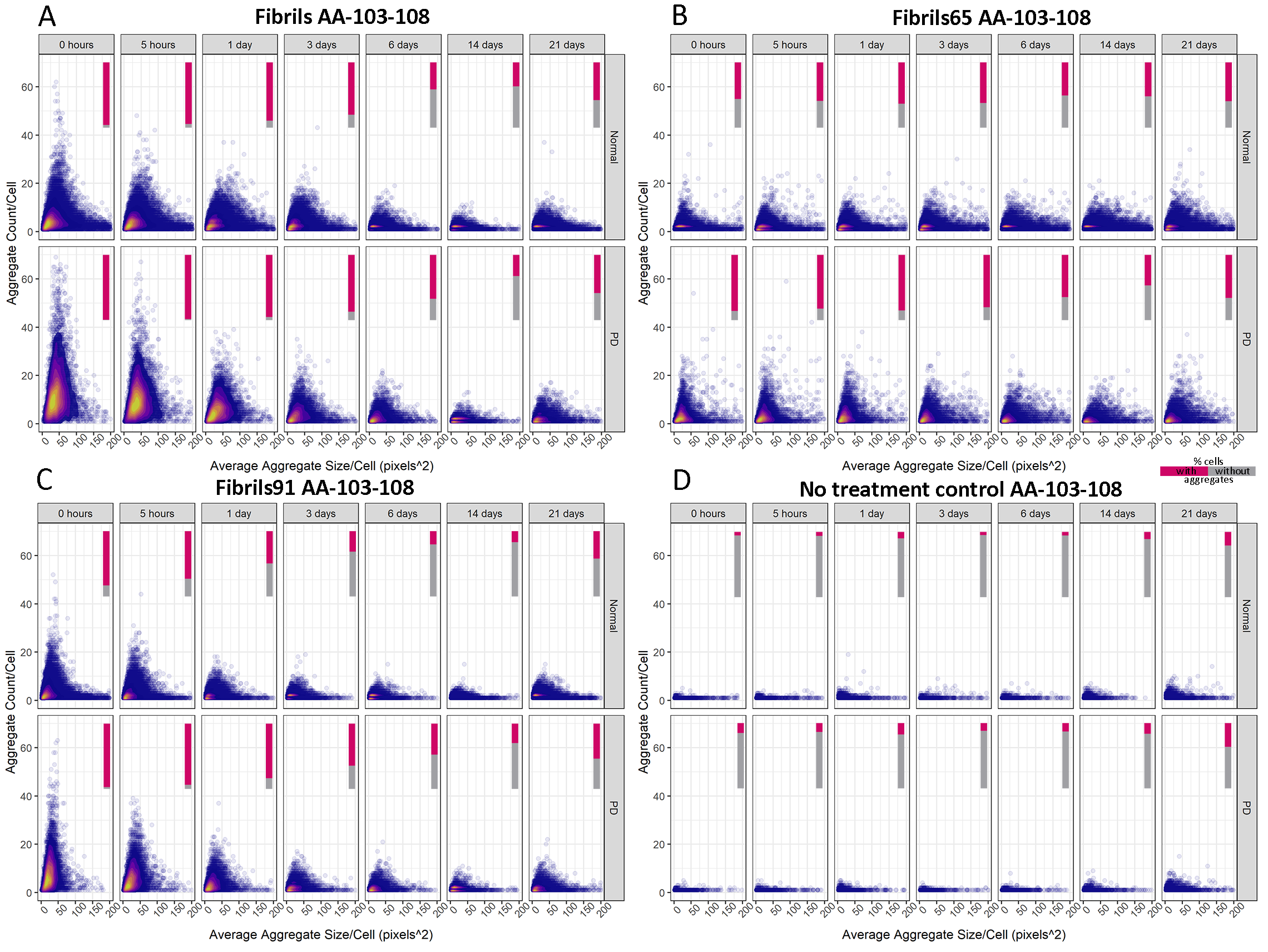

Supplement: S2 Fig — Cells without aggregates are excluded from density plots. Relative amounts of cells with aggregates represented in bar (% cells with aggregates in magenta, % cells without aggregates in grey). The colours represent a 2D kernel density estimation. It is scaled to 1 for each graph, with the bright yellow area displaying the highest density of cells. (TIF) [file pone.0277658.s002.tif]
