# Supplementary material for: Human pericytes degrade diverse α-synuclein aggregates
Source: PLoS One. 2022 Nov 18;17(11):e0277658. doi: 10.1371/journal.pone.0277658 (PMC9674377; doi:10.1371/journal.pone.0277658)

Fibrils and Ribbons
(used in Fig1F-J)

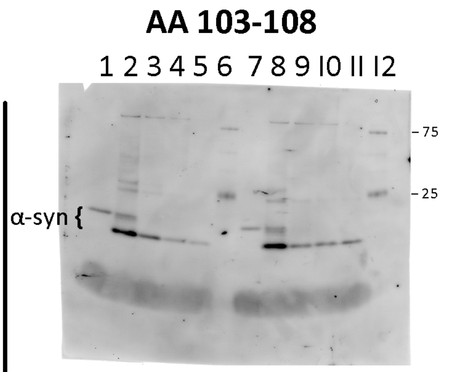

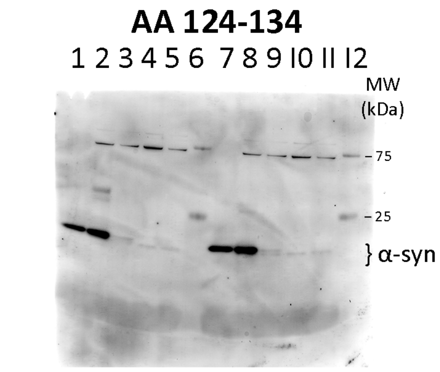

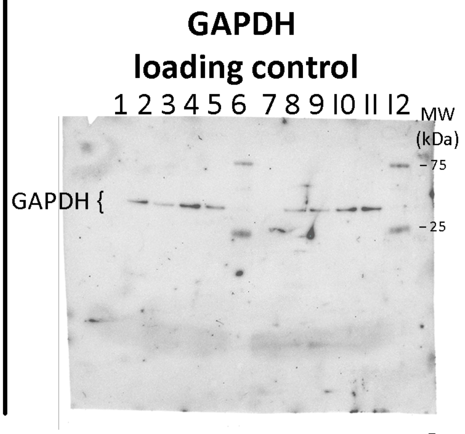

Lane 1	Fibrils alpha syn pure
Lane 2	Protein extract Fibrils 4h+4 hours
Lane 3	Protein extract Fibrils 4h+24 hours
Lane 4	Protein extract Fibrils 4h+14 days
Lane 5	Protein extract Fibrils 4h+21 days
Lane 6	Protein standard dual colour
Lane 7	Ribbons alpha syn pure
Lane 8	Protein extract Ribbons 4h+4 hours
Lane 9	Protein extract Ribbons 4h+24 hours
Lane 10	Protein extract Ribbons 4h+14 days
Lane 11	Protein extract Ribbons 4h+21 days
Lane 12	Protein standard dual colour

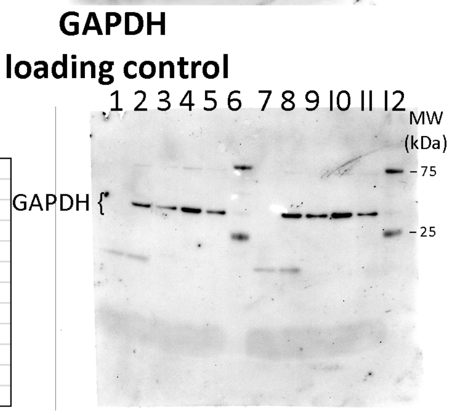

Fibrils65
(used in Fig1G-J)

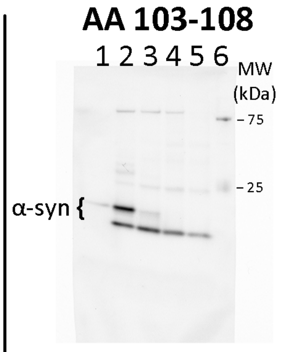

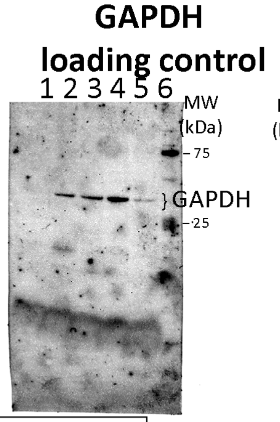

Lane 1	P65 alpha syn pure
Lane 2	Protein extract fibrils65 4h+4 hours
Lane 3	Protein extract fibrils65 4h+24 hours
Lane 4	Protein extract fibrils65 4h+14 days
Lane 5	Protein extract fibrils65 4h+21 days
Lane 6	Protein standard dual colour

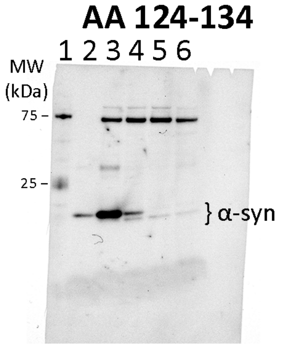

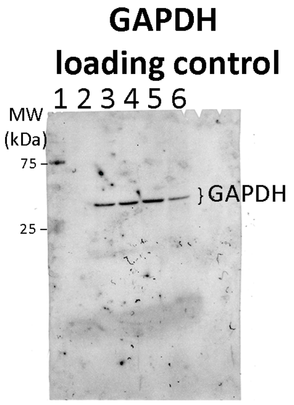

Lane 1	Protein standard dual colour
Lane 2	P65 alpha syn pure
Lane 3	Protein extract fibrils65 4h+4 hours
Lane 4	Protein extract fibrils65 4h+24 hours
Lane 5	Protein extract fibrils65 4h+14 days
Lane 6	Protein extract fibrils65 4h+21 days

α-syn monomer

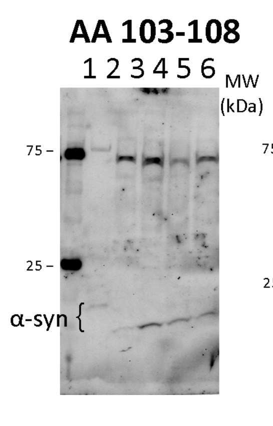

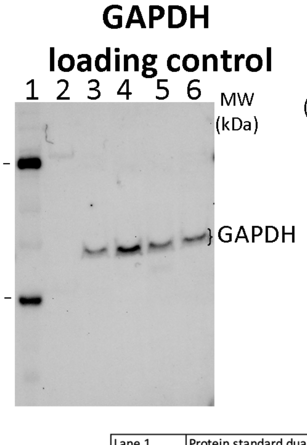

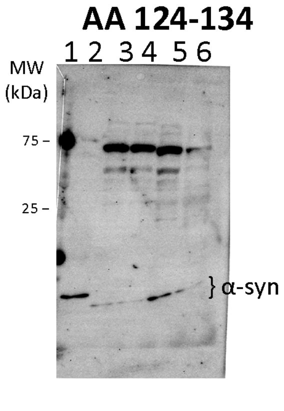

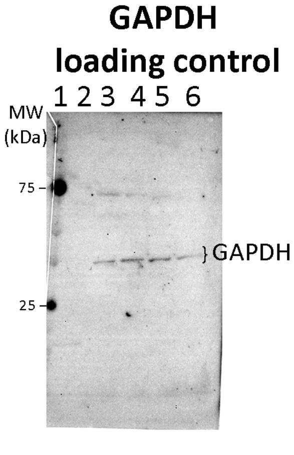

Lane 1	Protein standard dual colour
Lane 2	alpha syn monomer pure
Lane 3	Protein extract alpha syn monomer 4h+4 hours
Lane 4	Protein extract alpha syn monomer 4h+24 hours
Lane 5	Protein extract alpha syn monomer 4h+14 days
Lane 6	Protein extract alpha syn monomer 4h+21 hours

Fibrils91
(used in Fig1G-J)

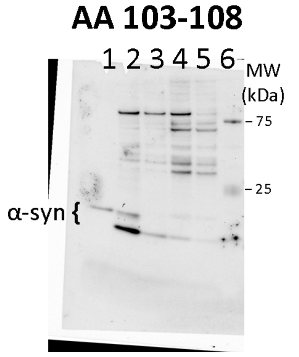

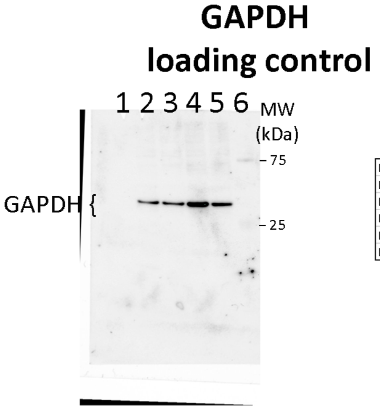

Lane 1	alpha syn pure
Lane 2	Protein extract fibrils91 4h+4 hours
Lane 3	Protein extract fibrils91 4h+24 hours
Lane 4	Protein extract fibrils91 4h+14 days
Lane 5	Protein extract P91 4h+21 days
Lane 6	Protein standard dual colour

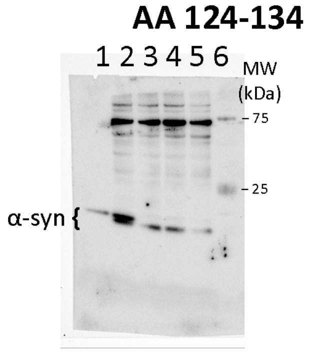

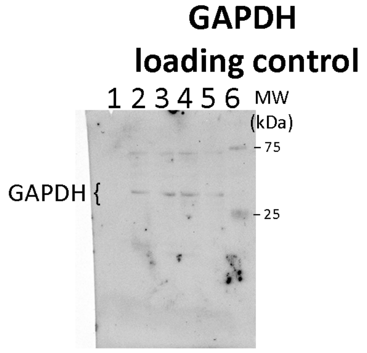

Fibrils110 & no treatment control
(used in Fig1G-J)

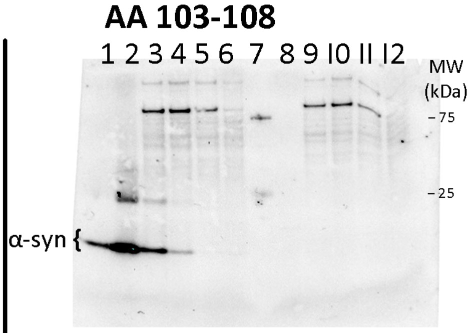

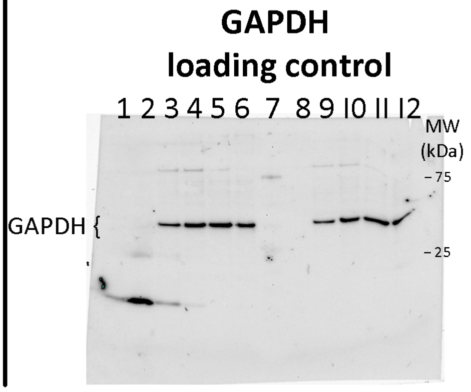

Lane 1	Fibrils110 alpha syn pure
Lane 2	Protein extract fibrils110 4h+4 hours
Lane 3	Protein extract fibrils110 4h+24 hours
Lane 4	Protein extract fibrils110 4h+14 days
Lane 5	Protein extract fibrils110 4h+21 days
Lane 6	Protein standard dual colour
Lane 7	PBS
Lane 8	Protein extract no treatment 4h+4 hours
Lane 9	Protein extract no treatment 4h+24 hours
Lane 10	Protein extract no treatment 4h+14 days
Lane 11	Protein extract no treatment 4h+21 days
Lane 12	Protein standard dual colour

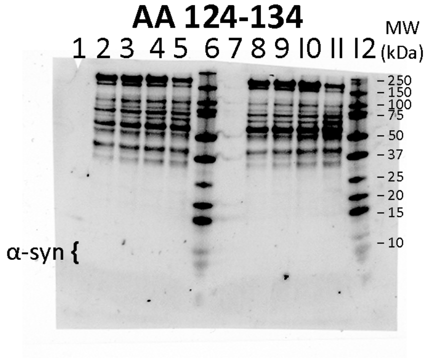

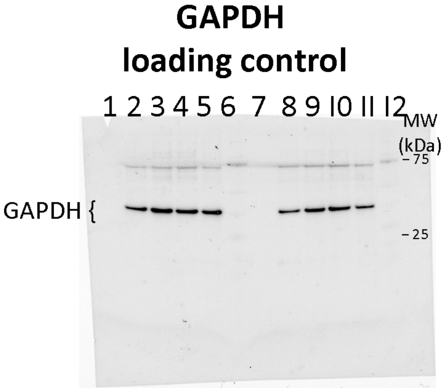

Supplement: S1 Raw images — Two blots used for each alpha synuclein strain. Each blot was labelled with α-syn antibodies (AA103-108 or AA124-134), imaged, stripped and relabelled for GAPDH. (PDF) [file pone.0277658.s003.pdf]
