# Supplementary material for: Human pericytes degrade diverse α-synuclein aggregates
Source: PLoS One. 2022 Nov 18;17(11):e0277658. doi: 10.1371/journal.pone.0277658 (PMC9674377; doi:10.1371/journal.pone.0277658)
Supplement: S1 File — (DOCX) [file pone.0277658.s011.docx]

##load appropriate packages. If have not installed, install by removing hashtags next to install.packages

##install.packages("tidyverse")

##install.packages("stringr")

library(tidyverse)

library(stringr)

library(dplyr)

##set working directory

setwd("T:\\Victor_reanalyze")

##folder inside wd that you want to concate excel sheets for

mydir = "nucleus_mask"

##load all excel files in and concate into one single dataframe

count_files <- list.files(path = mydir, pattern = "*.csv", full.names = TRUE)

count_meta <- purrr::map_df(count_files, function(x) {

data <- read.csv(x)

cbind(file_id = x, data)

})

##clean up dataframe

metaframe <- separate(count_meta, file_id, sep=" 6h", into = c("x", "timepoint"))

metaframe <- separate(metaframe, timepoint, sep="_", into = c("timepoint", "case"))

metaframe <- separate(metaframe, case, sep="0", into = c("case", "aggregate"))

metaframe = metaframe[-c(1,5)]

names(metaframe)[1] <- "Timepoint"

names(metaframe)[2] <- "Aggregate_Type"

names(metaframe)[3] <- "Case"

metaframe$Timepoint[metaframe$Timepoint =="-37day asyn no tag"] <- "37_days"

metaframe$Timepoint[metaframe$Timepoint =="-48h asyn no tag"] <- "2_days"

metaframe$Timepoint[metaframe$Timepoint =="-24h asyn no tag"] <- "1_day"

metaframe$Timepoint[metaframe$Timepoint =="-23day asyn no tag"] <- "23_days"

metaframe$Timepoint[metaframe$Timepoint =="-14day asyn no tag"] <- "14_days"

metaframe$Timepoint[metaframe$Timepoint =="-10day asyn no tag"] <- "10_days"

metaframe$Timepoint[metaframe$Timepoint =="-6day asyn no tag"] <- "6_days"

metaframe$Timepoint[metaframe$Timepoint =="-0h asyn no tag"] <- "0_hours"

metaframe$Timepoint[metaframe$Timepoint =="-5h asyn no tag"] <- "5_hours"

metaframe$Timepoint[metaframe$Timepoint =="-72h asyn no tag"] <- "3_days"

metaframe$Aggregate_Type[metaframe$Aggregate_Type =="B"] <- "fibrils"

metaframe$Aggregate_Type[metaframe$Aggregate_Type =="C"] <- "ribbons"

metaframe$Aggregate_Type[metaframe$Aggregate_Type =="D"] <- "p65"

metaframe$Aggregate_Type[metaframe$Aggregate_Type =="E"] <- "p91"

metaframe$Aggregate_Type[metaframe$Aggregate_Type =="F"] <- "p110"

metaframe$Aggregate_Type[metaframe$Aggregate_Type =="G"] <- "control"

metaframe$Case[metaframe$Case =="2"] <- "H189_P6-Control"

metaframe$Case[metaframe$Case =="3"] <- "H209_P7-Control"

metaframe$Case[metaframe$Case =="4"] <- "H238_P6-Control"

metaframe$Case[metaframe$Case =="5"] <- "PD52_P5-PD"

metaframe$Case[metaframe$Case =="6"] <- "PD78_P6-PD"

metaframe$Case[metaframe$Case =="7"] <- "PD65_P6-PD"

metaframe$Case[metaframe$Case =="8"] <- "PD63_P8-PD"

metaframe <- separate(metaframe, Case, sep= "-", into = c("Case", "Disease"))

metaframe <- separate(metaframe, Case, sep= "_", into = c("Case", "Passage"))

##set path to save file - change filename at the end to the name you want and remember to add filetype (.csv)

write.csv(metaframe, "U:\\BRAIN\\STAFF and STUDENT FOLDERS\\CURTIS GROUP\\Blake H\\Victor_aggregates\\outputty\\august_2021_metaframes\\nucleus_mask.csv", row.names = TRUE)

setwd("U:\\BRAIN\\STAFF and STUDENT FOLDERS\\CURTIS GROUP\\Blake H\\Victor_aggregates\\outputty\\august_2021_metaframes")

ms_counts <- read.csv("ms_count.csv")

ms_int <- read.csv("ms_int.csv")

nucleus_mask <- read.csv("nucleus_mask.csv")

#Load ggplot2 and ggridges

library(ggplot2)

library(ggridges)

# Reorder Timepoints for correct overlapping geom_points

ms_counts$Timepoint <- factor(ms_counts$Timepoint, levels = c("0_hours", "5_hours", "1_day", "2_days", "3_days", "6_days", "10_days", "14_days", "23_days", "37_days"))

ms_counts <- ms_counts[order(as.integer(ms_counts$Timepoint), decreasing = FALSE),]

ms_counts$Aggregate_Type_f <- factor(ms_counts$Aggregate_Type, levels = c("fibrils", "ribbons", "p65", "p91", "p110", "control"))

ms_int$Timepoint <- factor(ms_int$Timepoint, levels = c("0_hours", "5_hours", "1_day", "2_days", "3_days", "6_days", "10_days", "14_days", "23_days", "37_days"))

ms_int <- ms_int[order(as.integer(ms_int$Timepoint), decreasing = FALSE),]

ms_int$Aggregate_Type_f <- factor(ms_int$Aggregate_Type, levels = c("fibrils", "ribbons", "p65", "p91", "p110", "control"))

# Separate out groups by aggregate type for individual plots

ms_count_list <- split(ms_counts, ms_counts$Aggregate_Type)

ms_int_list <- split(ms_int, ms_int$Aggregate_Type)

## Plotting out counts over timepoints - Single aggregate for each

setwd("U:\\BRAIN\\STAFF and STUDENT FOLDERS\\CURTIS GROUP\\Blake H\\Victor_aggregates\\outputty\\august_2021_metaframes\\figures")

ms_fibrils <- ggplot(ms_count_list[["fibrils"]], aes(x=Count, y=Timepoint, fill = Disease)) + stat_density_ridges(rel_min_height = 0.01, scale = 2, quantile_lines = TRUE, quantiles = 2, alpha = .5) + facet_wrap(~Disease) + theme_bw() + ggtitle("Mouse Fibrils") + theme(plot.title = element_text(hjust = 0.5)) + coord_cartesian(xlim = c(0,40)) + scale_y_discrete(limits=rev) + scale_fill_manual(values = c("blue", "red"))

ggsave("ms_fibrils.png")

ms_ribbons <- ggplot(ms_count_list[["ribbons"]], aes(x=Count, y=Timepoint, fill = Disease)) + stat_density_ridges(rel_min_height = 0.01, scale = 2, quantile_lines = TRUE, quantiles = 2, alpha = .5) + facet_wrap(~Disease) + theme_bw() + ggtitle("Mouse Ribbons") + theme(plot.title = element_text(hjust = 0.5)) + coord_cartesian(xlim = c(0,40)) + scale_y_discrete(limits=rev) + scale_fill_manual(values = c("blue", "red"))

ggsave("ms_ribbons.png")

ms_p65 <- ggplot(ms_count_list[["p65"]], aes(x=Count, y=Timepoint, fill = Disease)) + stat_density_ridges(rel_min_height = 0.01, scale = 2, quantile_lines = TRUE, quantiles = 2, alpha = .5) + facet_wrap(~Disease) + theme_bw() + ggtitle("Mouse P65") + theme(plot.title = element_text(hjust = 0.5)) + coord_cartesian(xlim = c(0,40)) + scale_y_discrete(limits=rev) + scale_fill_manual(values = c("blue", "red"))

ggsave("ms_p65.png")

ms_p91 <- ggplot(ms_count_list[["p91"]], aes(x=Count, y=Timepoint, fill = Disease)) + stat_density_ridges(rel_min_height = 0.01, scale = 2, quantile_lines = TRUE, quantiles = 2, alpha = .5) + facet_wrap(~Disease) + theme_bw() + ggtitle("Mouse P91") + theme(plot.title = element_text(hjust = 0.5)) + coord_cartesian(xlim = c(0,40)) + scale_y_discrete(limits=rev) + scale_fill_manual(values = c("blue", "red"))

ggsave("ms_p91.png")

ms_p110 <- ggplot(ms_count_list[["p110"]], aes(x=Count, y=Timepoint, fill = Disease)) + stat_density_ridges(rel_min_height = 0.01, scale = 2, quantile_lines = TRUE, quantiles = 2, alpha = .5) + facet_wrap(~Disease) + theme_bw() + ggtitle("Mouse P110") + theme(plot.title = element_text(hjust = 0.5)) + coord_cartesian(xlim = c(0,40)) + scale_y_discrete(limits=rev) + scale_fill_manual(values = c("blue", "red"))

ggsave("ms_p110.png")

ms_control <- ggplot(ms_count_list[["control"]], aes(x=Count, y=Timepoint, fill = Disease)) + stat_density_ridges(rel_min_height = 0.01, scale = 2, quantile_lines = TRUE, quantiles = 2, alpha = .5) + facet_wrap(~Disease) + theme_bw() + ggtitle("Mouse Control") + theme(plot.title = element_text(hjust = 0.5)) + coord_cartesian(xlim = c(0,40)) + scale_y_discrete(limits=rev) + scale_fill_manual(values = c("blue", "red"))

ggsave("ms_control.png")

## Plotting out counts over timepoints - All aggregates combined onto one graph

ggplot(ms_counts, aes(x=Count, y=Timepoint, color = Aggregate_Type, fill = Aggregate_Type)) + stat_density_ridges(rel_min_height = 0.01, scale = 1, alpha = .2) + facet_wrap(~Disease) + coord_cartesian(xlim = c(0,40)) + scale_y_discrete(limits=rev) + theme_bw() + ggtitle("Mouse Aggregates") + theme(plot.title = element_text(hjust = 0.5))

ggsave("ms_combo_aggregates.png")

## Find summary statistics for PD normalized Timepoint plots

ms_summary_case_counts <- ms_counts %>%

group_by(Timepoint, Aggregate_Type, Case) %>%

summarise(

n = n(),

mean = mean(Count),

sd = sd(Count)

)

write.csv(ms_summary_case_counts, "U:\\BRAIN\\STAFF and STUDENT FOLDERS\\CURTIS GROUP\\Blake H\\Victor_aggregates\\outputty\\august_2021_metaframes\\ms_summary_case_counts.csv", row.names = TRUE)

ms_summary_disease_counts <- ms_counts %>%

group_by(Timepoint, Aggregate_Type, Disease) %>%

summarise(

n = n(),

mean = mean(Count),

sd = sd(Count)

)

write.csv(ms_summary_disease_counts, "U:\\BRAIN\\STAFF and STUDENT FOLDERS\\CURTIS GROUP\\Blake H\\Victor_aggregates\\outputty\\august_2021_metaframes\\ms_summary_disease_counts.csv", row.names = TRUE)

ms_summary_case_int <- ms_int %>%

group_by(Timepoint, Aggregate_Type, Case) %>%

summarise(

n = n(),

mean = mean(IntDen),

sd = sd(IntDen)

)

write.csv(ms_summary_case_int, "U:\\BRAIN\\STAFF and STUDENT FOLDERS\\CURTIS GROUP\\Blake H\\Victor_aggregates\\outputty\\august_2021_metaframes\\ms_summary_case_int.csv", row.names = TRUE)

ms_summary_disease_int <- ms_int %>%

group_by(Timepoint, Aggregate_Type, Disease) %>%

summarise(

n = n(),

mean = mean(IntDen),

sd = sd(IntDen)

)

write.csv(ms_summary_disease_int, "U:\\BRAIN\\STAFF and STUDENT FOLDERS\\CURTIS GROUP\\Blake H\\Victor_aggregates\\outputty\\august_2021_metaframes\\ms_summary_disease_int.csv", row.names = TRUE)
